# Human eyelid adipose tissue-derived Schwann cells promote regeneration of a transected sciatic nerve

**DOI:** 10.1038/srep43248

**Published:** 2017-03-03

**Authors:** Gangyang Wang, Lingling Cao, Yang Wang, Yingqi Hua, Zhengdong Cai, Jun Chen, Lulu Chen, Yuqing Jin, Lina Niu, Hua Shen, Yan Lu, Zunli Shen

**Affiliations:** 1 Department of Plastic and Reconstructive Surgery, Shanghai General Hospital, Shanghai Jiao Tong University School of Medicine, Shanghai, People’s Republic of China; 2 Department of Rehabilitation Medicine, Shanghai General Hospital, Shanghai Jiao Tong University School of Medicine, Shanghai, People’s Republic of China; 3 Department of Orthopaedics, Shanghai General Hospital, Shanghai Jiao Tong University School of Medicine, Shanghai, People’s Republic of China; 4 Plastic Surgery Hospital, Chinese Academy of Medical Sciences & Peking Union Medical College, Beijing, People’s Republic of China; 5 Department of Plastic and Reconstructive Surgery, Lanzhou Maternal and Child Health Care Hospital, Lanzhou, Gansu, People’s Republic of China

## Abstract

Schwann cells (SCs) can promote the regeneration of injured peripheral nerves while the clinical application is limited by donor site complications and the inability to generate an ample amount of cells. In this study, we have isolated human eyelid adipose-derived Schwann cells (hE-SCs) from human eyelid adipose tissue and identified the cell phenotype and function. Using immunofluorescence and H & E staining, we detected subtle nerve fibers and SCs in human eyelid adipose tissue. Immunofluorescence staining indicated that hE-SCs expressed glial markers, such as S100, p75NTR GFAP, Sox10 and Krox20. To explore whether hE-SCs promote the regeneration of injured peripheral nerves *in vivo,* a Balb/c-nu mice model was used in the study, and mice were randomly assigned to five groups: Matrigel; hE-SCs/P0; hE-SCs/P2; hE-FLCs/P2; and Autograft. After 12 weeks, functional and histological assessments of the regenerated nerves showed that sciatic nerve defect was more effectively repaired in the hE-SCs/P2 group which achieved 66.1 ± 6.5% purity, than the other three groups and recovered to similar level to the Autograft group. These results indicated that hE-SCs can promote the regeneration of injured peripheral nerve and the abundant, easily accessible supply of adipose tissue might be a promising source of SCs for peripheral nerve repair.

Peripheral nerve injuries (PNIs) are a common occurrence worldwide and often lead to chronic disabilities^1^,^2^. The best method for treating PNIs is directly suturing the nerve stump for nerve gaps of less than 5 mm^3^,^4^, and autologous nerve grafting for longer gaps is considered the gold standard^3^,^5^. However, nerve autografting is limited by the availability of graft material, the loss of sensation at the donor site and the possible formation of a painful neuroma^6^,^7^. Hence, new treatment approaches are required for managing PNIs. Over the past decade, tissue engineering techniques have been regarded as an alternative repair strategy for major PNIs^7^,^8^.

Schwann cells (SCs) are the principal seed cells in tissue engineering research and are crucially involved, both structurally and functionally, in peripheral nerve regeneration^9^,^10^,^11^,^12^. After peripheral nerves are transected, SCs are dedifferentiated and proliferated, forming Büngner bands, which are the scaffolds for the regenerating axons. When combined with tissue-engineered nerve conduits, SCs have tremendous potential to enhance nerve regeneration^13^,^14^. There are two main sources of autologous SCs: autologous nerves and various mesenchymal stem cells, such as umbilical cord mesenchymal stem cells (UMSC)^15^, bone marrow stromal cells (BMSC)^16^, adipose-derived mesenchymal stem cells (ASC)^17^, hair follicle stem cells^18^, etc. However, there are limitations in autologous nerve segment donor sites because of donor site morbidity, and there are limitations in the application of stem cells because of low induction rate and unstable differentiation results^19^. Therefore, the source of SCs has always been a challenge in biological medicine.

Adipose tissue forms in utero, in the peripartum period and throughout life. It plays a lot of roles, such as an arbiter of thermoregulation, a buffer against trauma and the cold, and a regulator of reproduction and satiety^20^. In mammals there are two main classes of adipose tissue that are separated into major histological divisions, white fat tissue (WAT) and brown fat tissue (BAT)^20^,^21^. WAT is used for energy storage whereas BAT generates heat and dissipates energy^20^,^22^. Adipose tissue exists in all the body parts, including the belly, the groin, the palms, the soles, the scalp, periarticular regions and the periorbital area^23^. There are tiny nerve fibers exist in adipose tissue which may be a promising source of SCs in peripheral nerve regeneration.

Previous studies have reported that cells harvested from different adipose tissue sites have been found to exhibit differences in characteristics^24^. For example, traits of cells isolated from omentum adipose tissue proliferate faster than those from the omentum^25^. For adipose stem cells (ASC) achieved efficiency, it is found to be higher in the abdomen than in the hip/thigh region^26^. Overall, most adipose tissues have been obtained from mesodermal-origin organs, while eyelid adipose tissues have been known to develop from ectodermal-origin, which have the same source of the neural crest^27^. Furthermore, eyelid adipose tissues are easily accessible and free of ethical complications. Hence, it is possible that hE-SCs may be an excellent candidate therapeutic cell type for PNIs.

There are two minimally invasive methods to acquire adipose tissue in plastic reconstruction surgeries: surgical operation and negative pressure suction^28^,^29^. Fortunately, both of these techniques acquire adipose tissue with few side effects in patients. Also, eyelid cosmetic surgery is more welcome than abdominoplasty in China. Therefore, it is feasible to get plenty of eyelid adipose tissue for PNIs patients in the clinic.

In this study, we have isolated human eyelid-derived Schwann cells (hE-SCs) from human eyelid adipose tissue and identified the cell phenotype and function for peripheral nerve repair both *in vitro* and *in vivo*.

## Results

### Detection and localization of Schwann cells and nerve fibers in human eyelid adipose tissue

**Figure 1:**
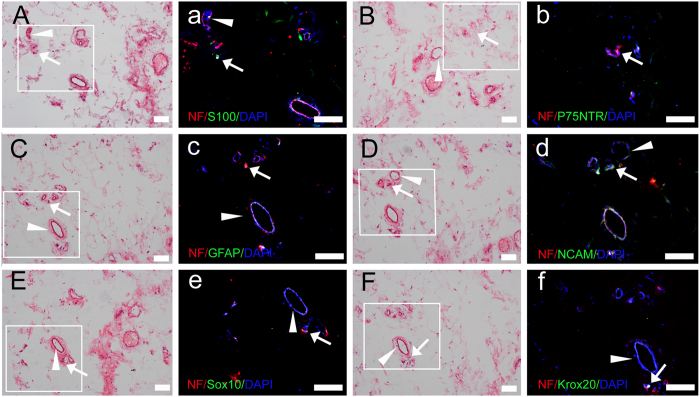
HE and immunofluorescence staining of Schwann cells and nerve fibers in human eyelid adipose tissue. (**A–F**) HE staining; (a–f) immunofluorescence staining. Nerve fibers (white arrow) were detected around the blood vessels (white triangle) in human eyelid adipose tissue. Bars = 100 μm.

To investigate the existence of SCs and nerve fibers in human eyelid adipose tissue, we performed HE and immunofluorescence staining. HE staining (Fig. 1A–F) revealed that some structures looked similar to nerve fibers around the blood vessels in human eyelid adipose tissue. Double staining for the axonal marker, NF-H and the SC markers, S100, P75NTR, GFAP, NCAM, Sox10 and Krox20 showed the spatial relationship between SCs and nerve fibers (Fig. 1a–f).

### Cell isolation and purification of hE-SCs

**Figure 2:**
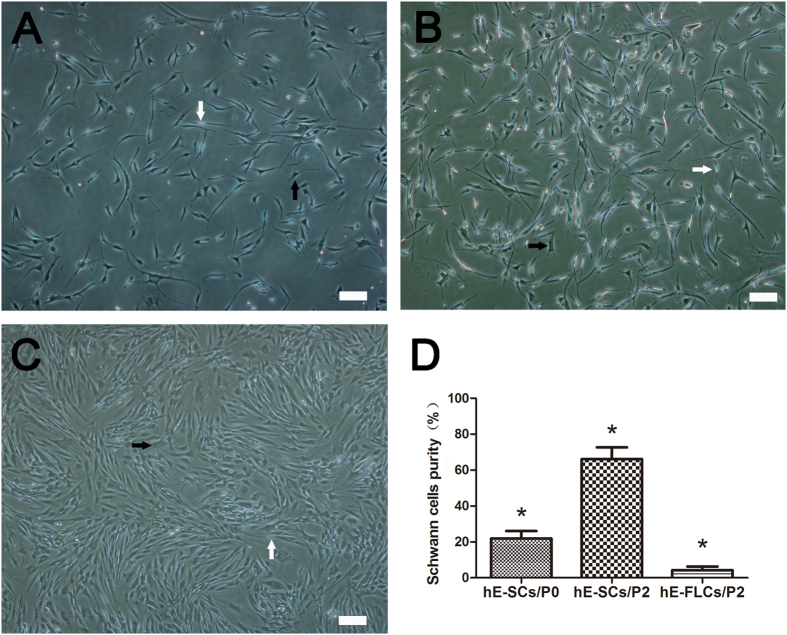
Phase-contrast photomicrographs of primary and secondary cultured cells. (**A**) After 24 h of primary culture, the cells developed two distinct shapes that represented two types of cells: hE-SCs(white arrows) and hE-FLCs(black arrows). (**B**) After two rounds of purification, the hE-SCs purity was improved. (**C**) The remnant hE-FLCs continued to rapidly proliferate and were passaged *in vitro*. (**D**) The purity of hE-SCs is shown as the mean ± S.D. from three independent experiments, *P < 0.05, significantly different compared to the hE-SCs/P2 group and the other two groups. (Scale bars = 100 μm).

Primary cells (i.e., hE-SCs/P0) were digested and then resuspended in Schwann cell culture media (SCCM). Most of the cells were attached to the bottom of the dishes within 48 h of culture and later developed into two distinct shapes (Fig. 2A). One cell shape was hE-SCs, which were characterized as a phase-refractile, bipolar or tripolar cell character having a small cytoplasm to nucleus ratio; the second cell shape was designated as fibroblast-like cells (hE-FLCs), which are characterized by a flat, poorly refractive polygon. The SC purity in the hE-SCs/P0 group was 21.9 ± 4.1%, which was determined using a cell counting method based on cell morphology differences. After two rounds of purification (about 12 days), the SC purity in the hE-SCs/P2 group was evaluated, and the results showed that the purity further increased to 66.1 ± 6.5% (Fig. 2B). The SC purity in the hE-FLCs/P2 group was 4.3 ± 1.9% (Fig. 2C). The SC purity in the hE-SCs/P2 group was significantly higher than the purities in the other two groups (*P < 0.05).

### Immunostaining characterization of hE-SCs

**Figure 3:**
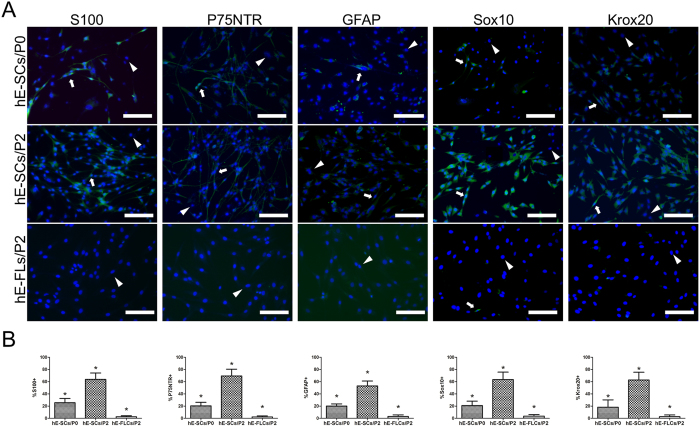
Immunocytochemical characterization of hE-SCs. (**A**) Immunofluorescent staining indicated that hE-SCs expressed S100, P75NTR, GFAP, Sox10 and Krox20 proteins. With the increased purity of the hE-SCs, the positive rates of S100, P75NTR, GFAP, Sox10 and Krox20 were also increased. hE-SCs were immuno-positive for S100, P75NTR, GFAP, Sox10 and Krox20 (white arrow), while hE-FLCs were negative for staining (white triangle). (Scale bars = 100 μm) (**B**) The percentage of hE-SCs expressing S100, P75NTR, GFAP, Sox10 and Krox20. Data are presented as the mean ± S.E.M, *P < 0.05 significantly different compared to the hE-SCs/P2 group and the other two groups.

As previously reported^30^,^31^,^32^, S100, P75NTR, GFAP, Sox10 and Krox20 have been used as markers for the characterization of SCs. As shown in Fig. 3A, hE-SCs with bipolar or tripolar morphologies were immuno-positive for S100, P75NTR, GFAP, Sox10 and Krox20; in contrast, hE-FLCs were negative for staining with these markers. The results showed that S100, P75NTR, GFAP, Sox10 and Krox20 were positive in primary cells (i.e., hE-SCs/P0), with rates of 25.56 ± 6.86, 20.56 ± 6.14, 20.06 ± 3.63, 20.86 ± 7.27 and 18.23 ± 11.97, respectively. After two rounds of purification, the positive rates in hE-SCs/P2 were increased to 63.68 ± 10.50, 69.43 ± 11.32, 53.16 ± 8.50, 63.46 ± 12.19 and 62.83 ± 12.61, respectively (Fig. 3B). The S100-, P75NTR-, GFAP-, Sox10- and Krox20-positive rates in the hE-SCs/P2 group were significantly higher than those in the other two groups (*P < 0.05).

### Electrophysiological evaluation of CMAP

**Figure 4:**
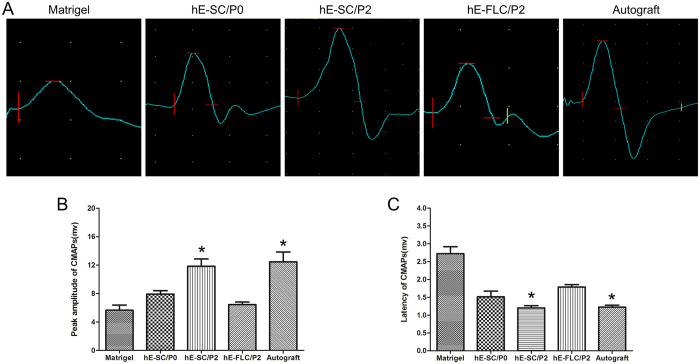
Electrophysiological evaluation of the injured site at 12 weeks after surgery. (**A**) Representative CMAP recordings of electrophysiological evaluation. Histograms show the peak (**B**) and latency periods (**C**) of the CMAPs. Data are presented as the mean ± standard error of three independent experiments, *P < 0.05; compared to the other three groups (Matrigel, hE-SCs/P0 and hE-FLs/P2).

We assessed motor function recovery using electrophysiological data (Fig. 4A). The peak amplitude of CMAPs in the hE-SCs/P2 group was significantly greater than those in the other three groups (*P < 0.05) and was comparable to that in the autologous nerve graft group (P > 0.05) (Fig. 4B). Moreover, the latency period of CMAPs in the hE-SCs/P2 group was significantly shorter than those in the other three groups (*P < 0.05) and recovered to a level close to that of the Autograft group (P > 0.05) (Fig. 4C). The results suggested that hE-SCs/P2 facilitate the effective restoration of nerve conductivity.

### Morphological evaluation of regenerated nerves

**Figure 5:**
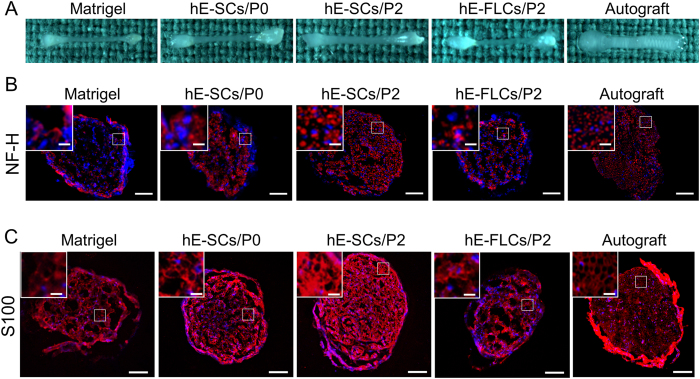
Macroscopic view and immunofluorescence staining of frozen sections of regenerated sciatic nerve tissue 12-weeks after surgery in the five groups. (**A**) Macroscopic view (**B**) anti-NF-H staining, and (**C**) anti-S100 staining. The white solid-line boxed areas were enlarged in the upper left inset. Images are representatives of the following groups: Matrigel, hE-SCs/P0. hE-SCs/P2 hE-FLCs/P2,and Autograft. Scale bars = 100 μm, scale bars in the upper left inset = 25 μm.

At 12 weeks after surgery, all the sciatic nerves succeeded in passing through the gap. Systematic histological assessments of the regenerated nerves were performed, including immunohistochemistry, toluidine blue staining and transmission electron microscopy. A macroscopic view of the regenerated nerves (Fig. 5A) shows complete sciatic nerve regeneration in all the groups from the proximal to distal nerve stumps. Obviously, the diameter of the regenerated nerves in the hE-SCs/P2 group was thicker than the diameters in the other three groups and was close to that in the Autograft group.

To better investigate the regeneration efficacy, sections of regenerated nerves were immunostained for NF-H (Fig. 5B) and S100 (Fig. 5C). The results showed that NF-H-positive axons exhibited a puncta-like phenotype and that S100-positive SCs formed ring shapes. In the hE-SCs/P2 group, the NF-H-positive axons and the S100-positive myelin sheath were densely and evenly distributed, whereas a decreased number of regenerated myelinated nerve fibers were noticeable in the other three groups (Matrigel, hE-SCs/P0 and hE-FLs/P2). In the Autograft group, most nerve fibers were regenerated along the autograft with an even distribution. The macroscopic view of the regenerated nerves showed that the nerve regeneration promoted by hE-SCs/P2 was similar to that in the Autograft group and stronger than that observed in the other three groups (Matrigel, hE-SCs/P0 and hE-FLs/P2).

**Figure 6:**
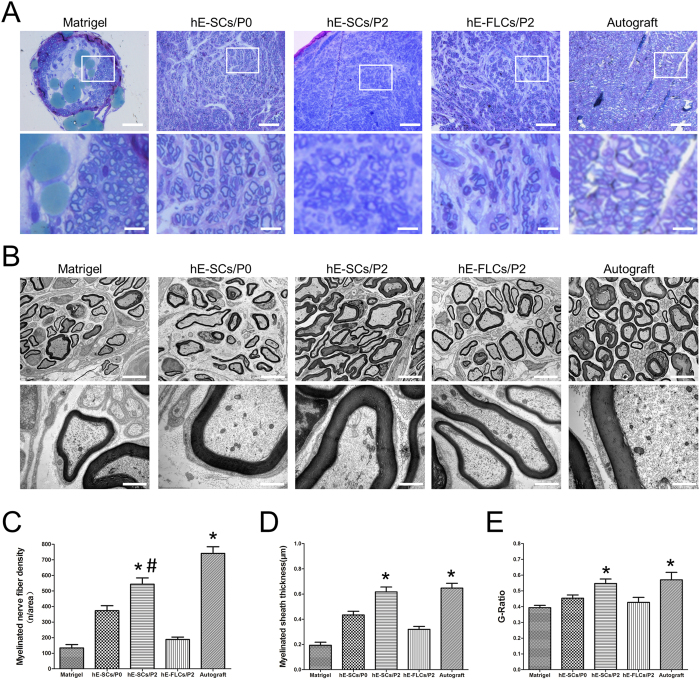
Morphology of the regenerated myelinated nerve fibers. (**A**) Toluidine blue staining of semi-thin sections from the middle of the nerve samples at 12 weeks after surgery. (Scale bars: 50 μm (upper panel) and 10 μm (lower panel). (**B**) TEM of ultrathin sections of the middle segment of the regenerated nerve samples at 12 weeks after surgery. The hE-SCs/P2 group showed poorly developed regenerated nerves composed of thin, dispersed myelinated or non-myelinated nerve fibers. (Scale bars: 10 μm (upper panel) and 0.5 μm (lower panel). Histograms showing the number of myelin sheath layers (**C**), the thickness of the regenerated myelin sheaths (**D**) and the G-ratios (axonal area/myelinated fiber area) (**E**). Data are shown as the mean ± SD, *P < 0.05, compared to the other three groups (Matrigel, hE-SCs/P0 and hE-FLs/P2). ^#^P < 0.05, compared to the hE-SCs/P2 group.

Toluidine blue-stained semi-thin sections from the middle of the nerve samples showed different sizes and amounts of regenerated neural fibers in each group. Clearly, the number of regenerated myelinated nerve fibers was higher in the hE-SCs/P2 group than in the other three groups (Matrigel, hE-SCs/P0 and hE-FLs/P2), but it was less than that in the autograft group (Fig. 6A and C). The densities of regenerated myelinated nerve fibers in each group were 133.67 ± 38.01 (n/area) for the Matrigel group, 373.33 ± 54.59 (n/area) for the hE-SCs/P0 group, 543 ± 70.92 (n/area) for the hE-SCs/P2 group, 188.33 ± 26.31 (n/area) for the hE-FLCs/P2 group, and 741.33 ± 73.36 (n/area) for the Autograft group. Transmission electron microscopy images showed that the regenerated axons consisted of many nerve fibers surrounded by a clear and electron-dense myelin sheath in each group. According to Fig. 6B and D, the regenerated myelinated axon areas in the hE-SCs/P2 group were thicker and larger than those in the other three groups (P < 0.05) and were similar to those in the autologous nerve graft group (P > 0.05). The myelin sheath thicknesses in each group were 0.19 ± 0.04 (μm) in the Matrigel group, 0.43 ± 0.05 (μm) in the hE-SCs/P0 group, 0.62 ± 0.07 (μm) in the hE-SCs/P2 group, 0.32 ± 0.04 (μm) in the hE-FLCs/P2 group, and 0.65 ± 0.07 (μm) in the Autograft group. The G-ratios (axonal area/myelinated fiber area) in each group were 0.39 ± 0.02 in the Matrigel group, 0.45 ± 0.04 in the hE-SCs/P0 group, 0.55 ± 0.04 in the hE-SCs/P2 group, 0.42 ± 0.05 in the hE-FLCs/P2 group, and 0.57 ± 0.08 in the Autograft group. These observations suggested that either the number of regenerated myelinated nerve fibers or the sheath thickness in the autograft or hE-SCs/P2 group was significantly greater than that in the other three groups.

### Evaluation of gastrocnemius muscle atrophy

**Figure 7:**
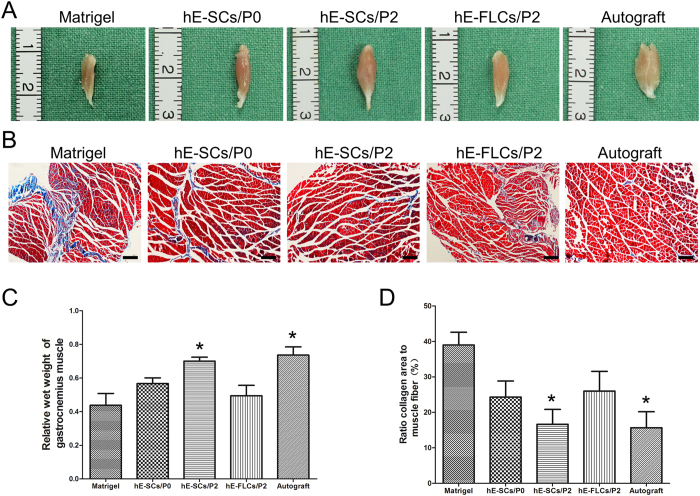
Wet weight and morphological analysis of the gastrocnemius muscle from the affected limb at 12 weeks after surgery. (**A**) Macroscopic view of the gastrocnemius muscles. (**B**) Masson trichrome staining of gastrocnemius muscle in five groups. Scale bars = 200 μm. (**C**) Quantification and statistical analysis of the wet weight ratios of gastrocnemius muscle. (**D**) Ratio of collagen area to the muscle fiber. Data are shown as the mean ± SD, *P < 0.05, compared to the other three groups (Matrigel, hE-SCs/P0 and hE-FLs/P2). Abbreviations: hE-SCs, human eyelid adipose-derived Schwann cells; hE-FLCs, human eyelid adipose-derived fibroblast-like cells.

After nerve transection, a decline in the neuromuscular innervation occurred: the denervated gastrocnemius muscles were atrophied and subsequently regained innervation after nerve regeneration. We measured the gastrocnemius muscle weights to assess the muscle atrophy and performed Masson’s trichrome staining to investigate the morphological changes in the gastrocnemius muscle. As shown in Fig. 7B there was little muscle fiber atrophy or collagen sedimentation in the hE-SCs/P2 group and the Autograft group, whereas there were more changes in the other three groups. The results indicated that the gastrocnemius muscle atrophy was less serious in the Autograft group or the hE-SCs/P2 group than in the other three groups, although the alleviation of muscle atrophy was greatest in the Autograft group (Fig. 7C and D).

## Discussion

Over the past decade, there has been extensive development of tissue engineering techniques that provide a new way for enhancing peripheral nerve regeneration and functional recovery^33^. Peripheral nerve repair with tissue-engineered technology requires a large quantity of seed cells. Autograft or allograft Schwann cells, olfactory ensheathing cells, adipose-derived stem cells (ASCs) (no differentiation to ectomesenchymal cells), and bone marrow mesenchymal stem cells (BMSCs) (no lure guide differentiation) have all shown certain effects on peripheral nerve regeneration^34^,^35^,^36^,^37^,^38^,^39^. Of these cellular approaches, autologous nerve tissue SCs are the most important seed cell for tissue engineering in nerve research^40^,^41^. However, the acquisition of autologous SCs still currently requires the sacrifice of autologous nerve tissue, which leads to sensory and autonomic dysfunction at the donor site^42^. The source of SCs is also an important issue that must be resolved^11^.

In the current study, we have shown that hE-SCs isolated from adult human eyelid adipose tissue were successfully used for bridging repair of 5-mm sciatic nerves. Our *in vitro* results demonstrated that hE-SC purification was up to 66.1% after two rounds of purification and that hE-SCs expressed neuronal and glial phenotypes, as well as S100, P75NTR, GFAP Sox10 and Krox20. Furthermore, *in vivo* studies showed that, hE-SCs were used to bridge a 5-mm sciatic nerve defect in Balb/c-nu mice. At 12 weeks after nerve grafting, systematic functional and histological assessments were performed to evaluate the regenerative capacity of hE-SCs. The recovery in the motor function of the injured hindlimb in hE-SCs/P2 group was close to that in autograft group without significant difference between each other, and prevailed over that in other three groups. Histological analysis showed that either the regenerated nerve or target gastrocnemius muscle achieved the similar reconstruction, between hE-SCs/P2 and autograft groups, and these similar results were significantly better than those in other three groups. All the results suggest that the easily accessible abundant supply of adipose tissue might be a potential source of SCs for peripheral nerve repair.

Adipose tissue is a mesodermally or ectodermally derived complex tissue that, besides adipocytes and pre-adipocytes, contains a stromal population, which includes smooth muscle cells, resident monocytes, lymphocytes, stem cells and endothelial and neuronal cells^43^. In this study, a unique scheme for isolating Schwann cells from adipose tissue was adopted to establish an optimal regenerative microenvironment. Adipose tissue is ubiquitous and uniquely expandable, and cells can be obtained from various types of adipose tissues, but those harvested from different tissue sites have been found to exhibit differences in characteristics^24^. Adipose tissue located in the abdomen, hip and thigh, are of mesodermal origin, while those located in the face are of ectodermal origin which have the same origin of the neural crest^25^. Previous studies have shown that the cells derived from facial adipose tissue expressed many neural stem cell-related proteins, unlike those derived from abdominal adipose tissues^27^. Therefore, human eyelid adipose-derived Schwann cells, owing to their neural properties, may represent a superior candidate to other cell types as seed cells for transplantation therapy of peripheral injuries.

First, we detected subtle nerve fibers and SCs in human eyelid adipose tissue through immunofluorescence and H&E staining. The results revealed the relationship between nerve fibers and SCs in human eyelid adipose tissue. Then, an efficient and stable method as previously reported^44^ was used to isolate and purify hE-SCs from adult human eyelid adipose tissue. After two rounds of purification, the purity of the hE-SCs reached 66.1%. Due to some limitations in the hE-SC purification protocol, the purity of hE-SCs reached only 60–70%. Previous reports have shown that anti-S100, anti-P75NTR, anti-GFAP anti-Sox10 and anti-Krox20 immunostaining can be used as markers for the characterization of SCs^17^,^45^. In our study, the positive rates in hE-SCs/P2 were 63.68 ± 10.50, 69.43 ± 11.32 and 53.16 ± 8.50. Flow cytometry was used to detect the expressions of P75NTR in hE-SCs/P2 cells; the results conformed with immunostaining (data not shown). The cell yields per unit of starting tissue also be calculated by cell counting using hematocytometer. At the end of the second purification, the final cell yield per unit of starting tissue was about 1.03 ± 0.03 × 10^6^ cells and it took about 12 days to generate enough cells for the *in vivo* transplantation. All the results proved that the cells isolated from adult human eyelid adipose tissue were Schwann cells and could be purified.

Subsequently, a well-established nude mice model of PNI was adapted to investigate the effects of hE-SCs *in vivo*. It was reported that Schwann cells could enhance motor neuron regeneration and improve evoked electrical potentials in the gastrocnemius muscle^46^. Moreover, undifferentiated ASCs have also been shown to enhance peripheral nerve repair^47^,^48^, which coincided with our results. Twelve weeks after nerve grafting, the electrophysiological evaluation and histological analyses of regenerated nerves was performed to compare the regenerative efficiency. Approximately 90% of the axons were regenerated across the 5-mm gap from the proximal to the distal part of the device in the hE-SCs/P2 group, and the axons recovered to a level close to that of the Autograft group. Electrophysiological assessments were performed to evaluate the effect of the injured nerve on gastrocnemius muscle activity. The peak amplitude and the latency period of CMAPs examinations are two important indices for the conduction of regenerated nerve function. Obviously, the hE-SCs/P2 promoted functional restoration more efficiently, which was accompanied by peripheral nerve regeneration.

Immunostaining demonstrated that the number of S100-positive myelin sheaths and NF-H-positive axons was significantly greater in the hE-SCs/P2 group than in the other three groups. This result could be due to the high purity of the hE-SCs, which more strongly promoted nerve regeneration through the formation of more myelin sheath relative to the other groups. To better evaluate the regeneration efficacy, we used toluidine blue staining and transmission electron microscopy to assess the semi-thin transverse sections of the middle segment of the nerve samples. The hE-SCs/P2 showed a stronger promotion of nerve regeneration than hE-SCs/P0 and hE-FLCs/P2. The number of myelinated nerve fibers and the myelin sheath thicknesses in the hE-SCs/P2 group was far greater than those in the other three groups.

Based on the immunofluorescence and TEM results, target muscle atrophy was further assessed through gastrocnemius muscle wet weight ratios and Masson’s trichrome staining. The denervated gastrocnemius muscles were atrophied and subsequently regained innervation after nerve regeneration^49^. The gastrocnemius muscle wet weight ratio was relatively higher in the hE-SCs/P2 group than in the other three groups, and the collagen fiber percentage was significantly lower in the hE-SCs/P2 group than in the other three groups.

In conclusion, this study demonstrates that human eyelid adipose tissue is a rich and promising source of SCs for the treatment of peripheral nerve injury. According to the above results, the study provides a safe and efficient method for isolating human eyelid adipose-derived Schwann cells (hE-SCs) from human eyelid adipose tissue. To date, this study is the first to demonstrate that SCs can be isolated from human eyelid adipose tissue, which is an important advancement in clinical applications to achieve functional recovery after peripheral nerve injury.

## Conclusion

We have isolated human eyelid adipose-derived Schwann cells (hE-SCs) from human eyelid adipose tissue and identified the cell phenotype and verified the efficacy for peripheral nerve repair both *in vitro* and *in vivo*. Our results indicated that hE-SCs were able to promote nerve regeneration and that the abundant supply of adipose tissue might be a promising source of SCs for peripheral nerve repair.

## Materials and Methods

### Isolation and Culture of human eyelid-derived Schwann cells (hE-SCs)

Human eyelid adipose samples were obtained with informed consent from six patients aged between 20 and 50 years undergoing eyelid cosmetic surgery, at Shanghai General Hospital. All experiments were approved by the institutional review board of Shanghai Jiao Tong Univerisity School of Medicine. Adipose tissues obtained from six patients were surgically dissected from the subcutaneous zone, cut into 1–2 mm^3^ pieces and washed twice with PBS. The tissues were digested with an enzymatic solution (100 μl per segment) prepared by dissolving DispaseII (Serva) and Collagenase NB4 (Serva, Germany) into DMEM at a concentration of 0.2% (0.27 U/ml). After 120 min at 37 °C, cell pellets were centrifuged at 1000 g for 5 min. Cell suspensions were cultured in 1 × 10^6^ cells/mL Schwann cell culture media (SCCM) composed of DMEM medium supplemented with 10% FBS (Hyclone, Australia), 1% penicillin-streptomycin (Invitrogen),10 ng/ml heregulin-β-1 (PeproTech, USA) and 2 μM forskolin (Sigma, USA)^50^. These primary cells (i.e hE-SCs/P0) were kept in a cell incubator in a humidified atmosphere of 5% CO_2_ at 37 °C.

As previously reported, hE-SCs were purified using a differential detachment method^44^. In brief, after primary cells were cultured for 48 hours, the culture medium was replaced with 0.05% (0.07 U/ml) Collagenase NB4 in DMEM medium supplemented with 10% FBS, 1% penicillin-streptomycin. After incubation for 20 min at 37 °C, the flasks were kept shaking horizontally for 1 min to release the lightly adhered cells (hE-SCs). Then the suspended cells were collected into a 50-ml conical tube (BD Falcon, USA) and centrifuged at 600 × g for 3 min. The tightly adhered cells (fibroblast-like cells, hE-FLCs) and lightly adhered cells (hE-SCs) were isolated based on their characteristics of adhesion to the culture dish. After two rounds of purification, we acquired higher purity hE-SCs (i.e hE-SCs/P2). And adherent hE-FLCs were cultured in SCCM at passage 2 (i.e hE-FLCs/P2).

The hE-SCs purity was determined on based on cell morphology. Cells that were phase-bright, spindle-shaped, bipolar, tripolar, and had a small cytoplasm to nucleus ratio were identified as hE-SCs, whereas cells with a flat polygonal shape and a prominent ovoid nucleus were identified as hE-FLCs. hE-SCs and hE-FLCs were counted in three photo areas randomly selected from each flask to obtain an average number. The purity of the hE-SCs was derived from the calculation of SC percentage with respect to the total number of counted cells.

### Immunofluorescence staining of hE-SCs

hE-SCs/P0, hE-SCs/P2,and hE-FLCs/P2 were trypsinized and replated on cover slides for immunostaining. The cells were fixed with 4% paraformaldehyde (Sigma) in PBS for 15 min. The fixed cells were washed three times with PBS for 5 min each, and then, the cells were blocked with 10% bovine serum albumin (Sigma, USA) in PBS for 30 min at room temperature, followed by three washes with PBS. The cells were then incubated with rabbit anti-glial fibrillary acidic protein (GFAP; 1:500; Abcam, Cambridge, UK), rabbit anti-P75NTR (1:500 diluted in PBS; Abcam, Cambridge, UK), rabbit anti-S100 (1:500; Dako, Glostrup, Denmark), rabbit anti-Sox10 (1:500 diluted in PBS; Abcam, Cambridge, UK), and rabbit anti-Krox10 (1:500 diluted in PBS; Abcam, Cambridge, UK) overnight at 4 °C. After being washed with PBS, the slides were treated with Alexa Fluor 488 goat anti-rabbit IgG (1:500; Invitrogen, USA) at 37 °C for 60 min. After they were washed with PBS, the cells were incubated with 1 M Hoechst 33343 (Sigma, USA) for 10 s. Labeled cells were examined with fluorescence microscopy (Olympus, Japan), and the images were recorded and processed with Image-Pro Plus (Media Cybernetics, USA).

### Sciatic nerve transection and implantation

Six- to eight-week-old female BALB/c-nu mice (Shanghai Slac Laboratory Animal Co., Ltd., Shanghai, China) weighing 24–30 g were anesthetized intraperitoneally with 0.5% chloral hydrate (6 ml/kg). All surgical procedures were performed using a microscope (Leica) and microsurgical instruments (Zhangjiagang Oscar Medical Instruments Co., Ltd., Zhangjiagang, China). After a 20-mm long operative incision was made on the dorsal skin of the left hind limb of each BALB/c-nu mice, the skin and hip muscles were separated, and the left sciatic nerves were carefully exposed and isolated. The exposed sciatic nerves were cut off to create 5-mm nerve defects, and two broken ends of the nerves were pulled 1 mm inside the silicone tubes with a length of 7.0 mm, and inner diameter of 0.9 mm (Shanghai Sincere Industry) and were sutured with 10–0 microsurgical sutures (Shanghai Pudong Jinhuan Medical Products) at a 1 mm margin of the corresponding ends of the conduit (7 mm). We used hE-SCs/P0, hE-SCs/P2, and hE-FLCs/P2 as seed cells to repair the 5-mm sciatic nerve defects in BALB/c-nu mice. Cells were resuspended in Matrigel (BD Biosciences, Franklin Lakes, NJ, USA) at a concentration of 1 × 10^6^ cells per 100 μl. Approximately 3.18 μl of the suspension (approximately 3.18 × 10^4^ cells) was used per animal. The animals were randomly assigned to the following five groups (n = 8 per group): (1) Matrigel: only Matrigel-filled silicon conduit; (2) hE-SCs/P0: primary cells (i.e., hE-SCs/P0, the hE-SCs purity was 21.9 ± 4.1%) filled silicon conduit; (3) hE-SCs/P2: high purity hE-SCs (i.e., hE-SCs/P2, the hE-SCs purity was 66.1 ± 6.5%) filled silicon conduit; (4) hE-FLCs/P2: (i.e., hE-FLCs/P2, the hE-SCs purity was 4.3 ± 1.9%) filled silicon conduit; (5) Autograft: the 5-mm transected nerve was re-implanted under a microscope. After surgery, all experimental animals were housed in a standard animal laboratory with free access to water and food. The electrophysiological evaluation and histological analyses of the regenerated nerves was performed at 12 weeks after the surgery.

All animal care and experimental studies were conducted according to the guidelines and approval of the Animal Investigation Committee of the Shanghai General Hospital, Shanghai Jiao Tong University School of Medicine. Male BALB/c athymic nude mice were bred and maintained at the animal center in Shanghai General Hospital (21 °C, 55% humidity, on a 12 h light/dark cycle).

### Electrophysiologic testing

At 12 weeks after surgery, electromyography was performed to evaluate the gastrocnemius muscle activity of the injured nerve. Following induction of anesthesia, the regenerated nerve and gastrocnemius muscle on the operated side were exposed. Double-hook stimulating electrodes were placed on the proximal end of the nerve trunk, and recording electrodes were penetrated into the middle of the gastrocnemius muscle on the injured side. The compound muscle action potential (CMAP) was recorded on the gastrocnemius muscle using a Medtronic Keypoint electromyography machine (Medtronic Inc., Minneapolis, MN, USA). The latency, amplitude, and nerve conduction velocity of the CMPAs were calculated. After electromyography, and the regenerated nerve and gastrocnemius muscle were taken for histological assessment.

### Histological assessment of regenerated nerve

After 12 weeks of surgery, regenerated nerves were harvested for toluidine blue staining and transmission electron microscopy (TEM). The middle segment of the regenerated nerves were fixed with 2.5% glutaraldehydein at 4 °C for 2 h and post-fixed with 1% osmium tetroxide for 2 h, dehydrated in the increasing concentrations of alcohol, and embedded with epoxy resin. Then semi-thin sections (1 μm thickness) of regenerated nerves were cut using an LKB-V ultramicrotome (LKB ProdukterB, Stockholm; Sweden), stained with 1% toluidine blue and photographed using an Axiocam digital camera (Zeiss, Oberkochen, Germany). The number of myelinated nerve fibers were counted (at a magnification of 100x) using a fluorescent microscope. Subsequently, the epoxy resin-embedded tissues were cut into ultra-thin sections (50 nm thickness), stained with lead citrate and observed under a transmission electron microscope (H7650, Hitachi, Tokyo, Japan). Five separate fields per slice were randomly selected to analyze myelin sheath thickness and G-Ratio.

Part of samples was used for immunohistochemistry. As previously described^51^, after the samples were fixed and blocked, the sections of peripheral nerves were incubated with rabbit anti-NF-H (1:200; Millipore, Boston, USA; for labeling regenerated axons) and rabbit anti-S100 (1:500; Dako, Glostrup, Denmark). The secondary antibody was Alexa Fluor 555 goat anti-rabbit IgG (1:1000; Invitrogen, Carlsbad, CA, USA). Nuclei were counterstained with 4′,6-diamidino-2-phenylindole (DAPI; 1:500, Invitrogen). After immunofluorescence staining, all images were captured with a TCS SP5 confocal laser scanning microscope (Leica, Germany).

### Wet weight analysis and Masson’s trichrome staining of gastrocnemius muscle

After the nerve samples were removed, the animals were sacrificed and the gastrocnemius muscles were rapidly and completely resected from the injured and normal sides and weighed. The wet weight ratio of gastrocnemius muscle was calculated by comparing the weight of the gastrocnemius muscle on the left (experimental) side to that on the right (normal) side. Then, the gastrocnemius muscle samples of the injured side were fixed in 4% paraformaldehyde at 4 °C overnight, embedded in paraffin, cut into 5-mm sections, and stained with Masson’s trichrome. The images were captured by an E600 microscope (Nikon, Tokyo, Japan) at a magnification of 200x and the collagen fiber percentage was quantitatively analyzed using IPP version 6.0 software (Media Cybernetics).

### Adipose tissue double-staining

Formalin-fixed, OCT-embedded human eyelid adipose tissue specimens were cut into serial sections (10 μm). Cryostat sections (10 μm) were used for haematoxylin and eosin staining. Briefly, after a hydration process, the slides were stained with hematoxylin for 15 min and immersed in 1% hydrochloric acid in 75% ethanol for 30 s.Then, the slides were stained with eosin for 5 min. Immunofluorescent staining was performed as above protocols. Image acquisition was performed using a fluorescence microscope.

### Statistical analyses

Data were expressed as mean ± standard deviations (SD). Unpaired student’s *t*-test, and one-way ANOVA was used to compare the differences among all the groups. All statistical analyses were performed using the statistical software SPSS version 19.0 (SPSS Inc., Chicago, IL, USA). Statistical significance was accepted at a P value of <0.05.

## Additional Information

**How to cite this article:** Wang, G. *et al*. Human eyelid adipose tissue-derived Schwann cells promote regeneration of a transected sciatic nerve. *Sci. Rep.* **7**, 43248; doi: 10.1038/srep43248 (2017).

**Publisher's note:** Springer Nature remains neutral with regard to jurisdictional claims in published maps and institutional affiliations.

## References

[b1] Wiberg M. & Terenghi G. Will it be possible to produce peripheral nerves? Surg Technol Int 11, 303–310 (2003). 12931315

[b2] Noble J., Munro C. A., Prasad V. S. & Midha R. Analysis of upper and lower extremity peripheral nerve injuries in a population of patients with multiple injuries. J Trauma 45, 116–122 (1998). 9680023 10.1097/00005373-199807000-00025

[b3] Pabari A., Yang S. Y., Seifalian A. M. & Mosahebi A. Modern surgical management of peripheral nerve gap. J Plast Reconstr Aesthet Surg 63, 1941–1948 (2010). 20061198 10.1016/j.bjps.2009.12.010

[b4] Tang S. . The effects of gradients of nerve growth factor immobilized PCLA scaffolds on neurite outgrowth *in vitro* and peripheral nerve regeneration in rats. Biomaterials 34, 7086–7096 (2013). 23791502 10.1016/j.biomaterials.2013.05.080

[b5] Lundborg G. A 25-year perspective of peripheral nerve surgery: evolving neuroscientific concepts and clinical significance. J Hand Surg Am 25, 391–414 (2000). 10811744 10.1053/jhsu.2000.4165

[b6] Carriel V., Alaminos M., Garzon I., Campos A. & Cornelissen M. Tissue engineering of the peripheral nervous system. Expert Rev Neurother 14, 301–318 (2014). 24506662 10.1586/14737175.2014.887444

[b7] Lin M. Y., Manzano G. & Gupta R. Nerve allografts and conduits in peripheral nerve repair. Hand Clin 29, 331–348 (2013). 23895714 10.1016/j.hcl.2013.04.003

[b8] Jiang X., Lim S. H., Mao H. Q. & Chew S. Y. Current applications and future perspectives of artificial nerve conduits. Exp Neurol 223, 86–101 (2010). 19769967 10.1016/j.expneurol.2009.09.009

[b9] Bunge R. P. Expanding roles for the Schwann cell: ensheathment, myelination, trophism and regeneration. Curr Opin Neurobiol 3, 805–809 (1993). 8260833 10.1016/0959-4388(93)90157-t

[b10] Bhatheja K. & Field J. Schwann cells: origins and role in axonal maintenance and regeneration. Int J Biochem Cell Biol 38, 1995–1999 (2006). 16807057 10.1016/j.biocel.2006.05.007

[b11] Rodrigues M. C., Rodrigues A. J., Glover L. E., Voltarelli J. & Borlongan C. V. Peripheral nerve repair with cultured schwann cells: getting closer to the clinics. ScientificWorldJournal 2012, 413091 (2012). 22701355 10.1100/2012/413091 PMC3373143

[b12] Yi S. . Regulation of Schwann cell proliferation and migration by miR-1 targeting brain-derived neurotrophic factor after peripheral nerve injury. Sci Rep 6, 29121 (2016). 27381812 10.1038/srep29121 PMC4933896

[b13] Li Q., Ping P., Jiang H. & Liu K. Nerve conduit filled with GDNF gene-modified Schwann cells enhances regeneration of the peripheral nerve. Microsurg 26, 116–121 (2006). 10.1002/micr.20192 16538638

[b14] Wakao S. . Long-term observation of auto-cell transplantation in non-human primate reveals safety and efficiency of bone marrow stromal cell-derived Schwann cells in peripheral nerve regeneration. Exp Neurol 223, 537–547 (2010). 20153320 10.1016/j.expneurol.2010.01.022

[b15] Xiao Y. Z. & Wang S. Differentiation of Schwannlike cells from human umbilical cord blood mesenchymal stem cells *in vitro*. Mol Med Rep 11, 1146–1152 (2015). 25369806 10.3892/mmr.2014.2840

[b16] Woodbury D., Schwarz E. J., Prockop D. J. & Black I. B. Adult rat and human bone marrow stromal cells differentiate into neurons. J Neurosci Res 61, 364–370 (2000). 10931522 10.1002/1097-4547(20000815)61:4<364::AID-JNR2>3.0.CO;2-C

[b17] Safford K. M., Safford S. D., Gimble J. M., Shetty A. K. & Rice H. E. Characterization of neuronal/glial differentiation of murine adipose-derived adult stromal cells. Exp Neurol 187, 319–328 (2004). 15144858 10.1016/j.expneurol.2004.01.027

[b18] Amoh Y. . Direct transplantation of uncultured hair-follicle pluripotent stem (hfPS) cells promotes the recovery of peripheral nerve injury. J Cell Biochem 110, 272–277 (2010). 20411592 10.1002/jcb.22534

[b19] Siciliano C. . The potential of GMP-compliant platelet lysate to induce a permissive state for cardiovascular transdifferentiation in human mediastinal adipose tissue-derived mesenchymal stem cells. Biomed Res Int 2015, 162439 (2015). 26495284 10.1155/2015/162439 PMC4606096

[b20] Berry D. C., Stenesen D., Zeve D. & Graff J. M. The developmental origins of adipose tissue. Development 140, 3939–3949 (2013). 24046315 10.1242/dev.080549 PMC3775412

[b21] Berry R. & Rodeheffer M. S. Characterization of the adipocyte cellular lineage *in vivo*. Nat Cell Biol 15, 302–308 (2013). 23434825 10.1038/ncb2696 PMC3721064

[b22] Cypess A. M. . Identification and importance of brown adipose tissue in adult humans. N Engl J Med 360, 1509–1517 (2009). 19357406 10.1056/NEJMoa0810780 PMC2859951

[b23] Gesta S., Tseng Y. H. & Kahn C. R. Developmental origin of fat: tracking obesity to its source. Cell 131, 242–256 (2007). 17956727 10.1016/j.cell.2007.10.004

[b24] van Harmelen V., Röhrig K. & Hauner H. Comparison of proliferation and differentiation capacity of human adipocyte precursor cells from the omental and subcutaneous adipose tissue depot of obese subjects. Metabolism 53, 632–637 (2004). 15131769 10.1016/j.metabol.2003.11.012

[b25] Kim S. H. . Character comparison of abdomen-derived and eyelid-derived mesenchymal stem cells. Cell Proliferat 46, 291–299 (2013). 10.1111/cpr.12027 PMC6496585 23692088

[b26] Jurgens W. J. F. M. . Effect of tissue-harvesting site on yield of stem cells derived from adipose tissue: implications for cell-based therapies. Cell Tissue Res 332, 415–426 (2008). 18379826 10.1007/s00441-007-0555-7 PMC2386754

[b27] Zhou J. . 17β-Estradiol Protects Human Eyelid-Derived Adipose Stem Cells against Cytotoxicity and Increases Transplanted Cell Survival in Spinal Cord injury. Journal of Cellular and Molecular Medicine 18, 326–343 (2014). 24373095 10.1111/jcmm.12191 PMC3930419

[b28] Amit A. & Tuabin R. A simple plunger technique for negative pressure during fat harvesting. J Plast Reconstr Aesthet Surg 67, e105–e106 (2014). 24315668 10.1016/j.bjps.2013.10.034

[b29] Hubner N. F., Horch R. E., Polykandriotis E., Rau T. T. & Dragu A. A histopathologic and immunohistochemical study on liquification of human adipose tissue *ex vivo*. Aesthetic Plast Surg 38, 976–984 (2014). 25015550 10.1007/s00266-014-0371-x

[b30] Mauritz C., Grothe C. & Haastert K. Comparative study of cell culture and purification methods to obtain highly enriched cultures of proliferating adult rat Schwann cells. J Neurosci Res 77, 453–461 (2004). 15248300 10.1002/jnr.20166

[b31] Raff M. C. . Cell-type-specific markers for distinguishing and studying neurons and the major classes of glial cells in culture. Brain Res 174, 283–308 (1979). 385109 10.1016/0006-8993(79)90851-5

[b32] Vroemen M. & Weidner N. Purification of Schwann cells by selection of p75 low affinity nerve growth factor receptor expressing cells from adult peripheral nerve. J Neurosci Methods 124, 135–143 (2003). 12706843 10.1016/s0165-0270(02)00382-5

[b33] Kumar R. .. Adult skin-derived precursor Schwann cells exhibit superior myelination and regeneration supportive properties compared to chronically denervated nerve-derived Schwann cells. Exp Neurol 278, 127–142 (2016). 26854934 10.1016/j.expneurol.2016.02.006

[b34] Levi A. D. . The use of autologous Schwann cells to supplement sciatic nerve repair with a large gap - First in human experience. Cell Transplant (2015). 10.3727/096368915X690198 26610173

[b35] Kabiri M. . Neuroregenerative effects of olfactory ensheathing cells transplanted in a multi-layered conductive nanofibrous conduit in peripheral nerve repair in rats. J Biomed Sci 22, 35 (2015). 25986461 10.1186/s12929-015-0144-0 PMC4437686

[b36] Walocko F. M., Khouri R. J., Urbanchek M. G., Levi B. & Cederna P. S. The potential roles for adipose tissue in peripheral nerve regeneration. Microsurg 36, 81–88 (2016). 10.1002/micr.22480 PMC5089369 26773850

[b37] Wang Y. . Recellularized nerve allografts with differentiated mesenchymal stem cells promote peripheral nerve regeneration. Neurosci Lett 514, 96–101 (2012). 22405891 10.1016/j.neulet.2012.02.066

[b38] Buzoianu-Anguiano V. . The Morphofunctional Effect of the Transplantation of Bone Marrow Stromal Cells and Predegenerated Peripheral Nerve in Chronic Paraplegic Rat Model via Spinal Cord Transection. Neural Plast 2015, 389520 (2015). 26634157 10.1155/2015/389520 PMC4655076

[b39] Mimura T., Dezawa M., Kanno H., Sawada H. & Yamamoto I. Peripheral nerve regeneration by transplantation of bone marrow stromal cell-derived Schwann cells in adult rats. J Neurosurg 101, 806–812 (2004). 15540919 10.3171/jns.2004.101.5.0806

[b40] Stratton J. A. & Shah P. T. Macrophage polarization in nerve injury: do Schwann cells play a role? Neural Regen Res 11, 53–57 (2016). 26981078 10.4103/1673-5374.175042 PMC4774224

[b41] Lehmann H. C. & Hoke A. Use of engineered Schwann cells in peripheral neuropathy: Hopes and hazards. Brain Res 1638, 97–104 (2016). 26529642 10.1016/j.brainres.2015.10.040

[b42] Wang L., Sanford M. T., Xin Z., Lin G. & Lue T. F. Role of Schwann cells in the regeneration of penile and peripheral nerves. Asian J Androl 17, 776–782 (2015). 25999359 10.4103/1008-682X.154306 PMC4577590

[b43] Kokai L. E., Rubin J. P. & Marra K. G. The Potential of Adipose-Derived Adult Stem Cells as a Source of Neuronal Progenitor Cells. Plast Reconstr Surg 116, 1453–1460 (2005). 16217495 10.1097/01.prs.0000182570.62814.e3

[b44] Jin Y. Q., Liu W., Hong T. H. & Cao Y. Efficient Schwann cell purification by differential cell detachment using multiplex collagenase treatment. J Neurosci Methods 170, 140–148 (2008). 18295342 10.1016/j.jneumeth.2008.01.003

[b45] Keilhoff G., Goihl A., Langnase K., Fansa H. & Wolf G. Transdifferentiation of mesenchymal stem cells into Schwann cell-like myelinating cells. Eur J Cell Biol 85, 11–24 (2006). 16373171 10.1016/j.ejcb.2005.09.021

[b46] di Summa P. G. . Long-term *in vivo* regeneration of peripheral nerves through bioengineered nerve grafts. Neuroscience 181, 278–291 (2011). 21371534 10.1016/j.neuroscience.2011.02.052

[b47] Mohammadi R., Azizi S., Delirezh N., Hobbenaghi R. & Amini K. Comparison of beneficial effects of undifferentiated cultured bone marrow stromal cells and omental adipose-derived nucleated cell fractions on sciatic nerve regeneration. Muscle Nerve 43, 157–163 (2011). 21254077 10.1002/mus.21895

[b48] Shen C. C., Yang Y. C. & Liu B. S. Peripheral nerve repair of transplanted undifferentiated adipose tissue-derived stem cells in a biodegradable reinforced nerve conduit. J Biomed Mater Res A 100, 48–63 (2012). 21972223 10.1002/jbm.a.33227

[b49] Raimondo S. . Ghrelin: a novel neuromuscular recovery promoting factor? Int Rev Neurobiol 108, 207–221 (2013). 24083436 10.1016/B978-0-12-410499-0.00008-3

[b50] Casella G. T., Bunge R. P. & Wood P. M. Improved method for harvesting human Schwann cells from mature peripheral nerve and expansion *in vitro*. Glia 17, 327–338 (1996). 8856329 10.1002/(SICI)1098-1136(199608)17:4<327::AID-GLIA7>3.0.CO;2-W

[b51] Zhang T. . Natural product pectolinarigenin inhibits osteosarcoma growth and metastasis via SHP-1-mediated STAT3 signaling inhibition. Cell Death and Disease 7, e2421 (2016). 27735939 10.1038/cddis.2016.305 PMC5133974

